# Higher association of coronary artery calcification with non-alcoholic fatty liver disease than with abdominal obesity in middle-aged Korean men: the Kangbuk Samsung Health Study

**DOI:** 10.1186/s12933-015-0253-9

**Published:** 2015-07-15

**Authors:** Min-Kyung Lee, Hye-Jeong Park, Won Seon Jeon, Se Eun Park, Cheol-Young Park, Won-Young Lee, Ki-Won Oh, Sung-Woo Park, Eun-Jung Rhee

**Affiliations:** Department of Endocrinology and Metabolism, Seonam University Myongji Hospital, Goyang, Korea; Department of Endocrinology and Metabolism, Kangbuk Samsung Hospital, Sungkyunkwan University School of Medicine, 108 Pyungdong, Jongro-ku, Seoul, Korea

**Keywords:** Coronary artery calcification, Non-alcoholic fatty liver disease, Abdominal obesity

## Abstract

**Background:**

It is uncertain whether non-alcoholic fatty liver disease (NAFLD) or abdominal obesity is more associated with atherosclerosis. The aim of this study was to determine whether NAFLD or abdominal obesity is more strongly associated with subclinical atherosclerosis represented by coronary artery calcification (CAC).

**Methods:**

A total of 21,335 male participa nts in a health screening program (mean age 41 years) were enrolled. Ultrasonographic measurements of fatty liver and multi-detector computed tomography were performed to determine the coronary artery calcium score (CACS). The presence of CAC was defined as CACS >0. Subjects were divided into four groups according to the presence or absence of NAFLD and/or abdominal obesity as assessed by waist-hip ratio (WHR) >0.9.

**Results:**

The presence of CAC was detected in 2,385 subjects (11.2%). The proportion of subjects with CAC was highest in the abdominal obesity only group (23.2%). After adjustment for age, diabetes history, hypertension, cigarette smoking, and physical inactivity, the odds ratio (OR) for CAC was the highest in the group with both abnormalities [1.465 (1.324–1.623)]. The NAFLD only group showed significantly increased OR for CAC compared to that in the abdominal obesity only group [1.286 (1.151–1.436) vs. 1.076 (0.939–1.233)].

**Conclusion:**

Non-alcoholic fatty liver disease is more closely associated with CAC than abdominal obesity as assessed by the WHR. NAFLD could be considered an independent determinant of subclinical atherosclerosis as assessed by CAC.

## Background

Coronary artery disease (CAD) due to atherosclerosis is a leading cause of morbidity and mortality worldwide and continues to be a significant health problem and global burden [1]. Early detection of subclinical atherosclerosis is necessary to prevent progression towards overt CAD. Obesity is considered an independent risk factor for atherosclerosis and CAD [2, 3], and is also associated with other CAD risk factors including hypertension, diabetes, and dyslipidemia [4, 5]. Recent studies have concentrated on the localized distribution of body fat rather than overall obesity, and abdominal obesity has thus been indicated as a strong risk factor for CAD [6, 7].

Non-alcoholic fatty liver disease (NAFLD) has become an emerging public health concern that parallels the rise in metabolic syndrome and obesity [8, 9]. NAFLD is now recognized as the hepatic manifestation of metabolic syndrome and insulin resistance (IR) [8]. Recent studies suggest that NAFLD contributes to the development of subclinical atherosclerosis or cardiovascular disease and could be considered as a cardiovascular risk factor [10–13]. The risk assessment of NAFLD and abdominal obesity is clinically important for the application of both lifestyle and/or pharmacological therapies targeted to lower atherosclerosis. However, it is unclear whether NAFLD or abdominal obesity is more strongly associated with atherosclerosis.

Early detection of subclinical atherosclerosis is important to prevent overt CAD, and coronary artery calcification (CAC) is a useful marker of subclinical atherosclerosis [14]. CAC score (CACS) reflects the general cardiovascular burden of the arteries in our body as it correlates well with the atheromatous plaque burden in autopsy studies [15]. CAC scoring with multi-detector computed tomography (MDCT) is a useful and non-invasive tool for risk prediction of subclinical atherosclerosis in asymptomatic individuals and also correlated well with cardiovascular events and obesity [16].

Recent studies suggest the association of NAFLD with subclinical atherosclerosis assessed by CACS [17–23]. The aim of the present study was to determine whether NAFLD or abdominal obesity is more associated with subclinical atherosclerosis in apparently healthy Korean men. To answer this question, we assessed the risk of CAC in subjects divided into four groups according to the presence/absence of NAFLD as diagnosed by ultrasonography and presence/absence of abdominal obesity measured by the waist-hip ratio (WHR) status. We analyzed CAC in these groups to clarify whether NAFLD or abdominal obesity is associated with CAC.

## Methods

### Study design and study population

This cross-sectional retrospective study was a part of the Kangbuk Samsung Health Study, in which subjects participated in a medical health check program at the Health Promotion Center of Kangbuk Samsung Hospital, Sungkyunkwan University, Seoul, Korea. The purpose of this medical health check program was to promote the health of employees through regular checkups and to enhance early detection of existing diseases. Health checks included blood tests, anthropometry, and abdominal ultrasonography examination, and in some cases, the health checks included CAC scoring by MDCT. The CAC test is offered as part of the routine health check program and therefore there is no medical indication for performing the test.

Ultrasonography measurements of fatty liver and MDCT for CAC scoring were performed in 26,857 men in the medical checkup program between January 2010 and December 2011. We excluded subjects who had a self-reported history of heart attack and CAD including acute myocardial infarction, angina, or congestive heart failure; subjects who reported a daily alcohol intake ≥20 g; subjects having serologic evidence of viral hepatitis or other chronic liver disease; and subjects with any missing data. A total of 21,335 men were selected for analysis.

### Anthropometric and laboratory measurements

Blood pressure was measured using a standardized sphygmomanometer after 5 min of rest, according to the Hypertension Detection and Follow-up Program protocol [24]. Height and weight were measured twice and then averaged. The BMI was calculated by dividing the weight (kg) by the square of the height (m). The waist circumference (WC) was measured in the standing position at the levels of the umbilicus.

All subjects were examined after a 12-h overnight fast. We analyzed blood samples for aspartate aminotransferase, alanine aminotransferase, gamma-glutamyl transpeptidase, total serum cholesterol, serum triglycerides, serum high-density lipoprotein cholesterol, low density lipoprotein cholesterol, fasting blood glucose level, serum high-sensitivity C-reactive protein (hs-CRP), serum insulin level, and glycated hemoglobin A1c (HbA1c). Biochemical markers were measured using Bayer Reagent Packs on an automated chemistry analyzer (ADVIA 1650 Autoanalyzer; Bayer HealthCare, Tarrytown, NY, USA). Lipid profiles were measured via enzymatic colorimetric assay. Fasting blood glucose levels were measured using the hexokinase method. Serum hs-CRP levels were measured using a nephelometric assay with a BNII nephelometer (Dade Behring, Deerfield, IL, USA). Insulin resistance was measured using the homeostatic model for the assessment index-insulin resistance (HOMA-IR) and was obtained by applying the following formula: HOMA-IR = fasting insulin (IU/mL) × fasting blood glucose (mmol/L)/22.5 [25]. HbA1c was measured using the immunoturbidimetric assay with a Cobra Integra 800 automatic analyzer (Roche Diagnostics, Basel, Switzerland) with a reference value of 4.4–6.4% [26].

The presence of diabetes mellitus was determined from answers in the self-questionnaire and using the diagnostic criteria of the American Diabetes Association [27]. The presence of hypertension was defined as blood pressure (BP) ≥140/90 mm Hg or presently taking anti-hypertensive medication, according to the criteria recommended by the seventh report of the Joint National Committee on prevention, detection, evaluation, and treatment of high BP [28]. Smoking status was determined using the questionnaire. A smoker was defined as a subject who had ever smoked at least five packs of cigarettes in his life. Exercising was defined as regular exercise of moderate intensity every week.

### Measurement of coronary artery calcium score

MDCT for coronary calcium scoring was undertaken using a 64-slice, spiral computed tomography scan (GE Health Care, Tokyo, Japan). CACS were expressed in Agaston units and the presence of CAC was defined by CACS >0 [29].

### Diagnosis of NAFLD and abdominal obesity

Abdominal ultrasonography (ASPEN; Acuson, Pennsylvania, USA) was performed by one of three radiologists using a 3.5 MHz probe to evaluate the presence of hepatic steatosis in all subjects. The diagnosis of fatty liver was made based on the following criteria [30, 31]: a diffuse hyperechoic echotexture, hepatorenal echo contrast in reference to the cortex of the right kidney, and vascular blurring and deep-echo attenuation. When making the diagnosis of NAFLD, the results of the liver function test were not taken into consideration and liver tissue was not examined.

Body composition measurements were carried out by segmental bioelectric impedance, using eight tactile electrodes according to the manufacturer’s instructions (InBody 3.0, Biospace Co. Ltd, Seoul, Korea). Lean mass (kg), fat mass (kg), percent fat mass (%), and WHR as a marker of abdominal obesity, were measured. Abdominal obesity, known clinically as central obesity, was assessed by WHR >0.90 for men using the measurement of body composition [32].

### Ethics

The participants provided written informed consent for the use of their health screening data for this research. The design, protocol, and consent procedure of this study were reviewed and approved by the Institutional Review Board (IRB) of Kangbuk Samsung Hospital and are in accordance with the Helsinki Declaration of 1975. After the review and acceptance of the study protocol by the IRB, a specific dataset for this study was released to the data management group of the KSHS after deleting the personal information of the participants.

### Statistical analysis

All data are presented as mean ± standard deviation, median (interquartile range) or percentage and were analyzed using IBM SPSS version 18.0 (IBM, Armonk, NY, USA). We divided subjects into four groups according to individual NAFLD and abdominal obesity status. Comparison of continuous variables between the groups was performed by one-way analysis of variance test and post hoc analyses with the Tukey’s b method. Nonparametric comparisons of the medians between the groups were performed using Kruskal–Wallis H test and Mann–Whitney U test for post hoc analyses with Bonferroni correction. We used Chi square tests for the categorical variables. Analysis of covariance test was used to adjust for age.

As CACS values were extremely skewed, logarithmized CACS + 1 was used for the comparison of between the groups. CACSs were dichotomized as presence of CACS >0 versus absence of CACS = 0 for binary logistic regression analysis. Multivariate logistic regression analyses were performed to assess the association between groups with CAC (CACS >0) while controlling for potential confounding variables included in the model. Covariates in the multivariable model, chosen for clinical importance as well as statistical significance included age, diabetes, hypertension, cigarette smoking, and physical inactivity. Asymptomatic significances (two-sided tests) are reported. Significance was defined as *p* < 0.05. Odds ratios (ORs) and 95% confidence intervals (CIs) were obtained.

## Results

### General characteristics of the participants

A total of 21,335 men (mean age 41 years; range 23–88 years) were divided into four groups according to the presence or absence of NAFLD and/or abdominal obesity as assessed by the WHR (Table 1) as follows: (1) subjects without either abnormality (n = 9,052; 42.4%); (2) subjects with abdominal obesity only (n = 2,220; 10.4%); (3) subjects with NAFLD only (n = 4,859; 22.8%); and (4) subjects with both abnormalities (n = 5,204; 24.5%).

**Table 1:** Baseline characteristics between the groups according to nonalcoholic fatty liver disease and abdominal obesity status

	Total population	NAFLD (−) abdominal obesity (−)	NAFLD (−) abdominal obesity (+)	NAFLD (+) abdominal obesity (−)	NAFLD (+) abdominal obesity (+)	*p*
N (%)	21,335 (100)	9,052 (42.4)	2,220 (10.4)	4,859 (22.8)	5,204 (24.5)	
Age (year)	40.8 ± 7.3	39.5 ± 6.7^a^	45.1 ± 9.4	39.4 ± 5.8^a^	42.3 ± 7.6	<0.001
SBP (mmHg)	114.9 ± 12.0	112.3 ± 11.4	116.1 ± 12.2	115.1 ± 11.5	118.8 ± 12.1	<0.001
DBP (mmHg)	74.5 ± 9.6	72.4 ± 9.0	77.7 ± 9.7	75.7 ± 9.5	76.65 ± 9.6	<0.001
FBS (mg/dL)	99.1 ± 16.8	95.9 ± 12.4	99.2 ± 14.2^a^	100.0 ± 18.5^a^	103.8 ± 21.0	<0.001
TC (mg/dL)	203.0 ± 31.2	196.2 ± 32.7	203.9 ± 34.7	206.2 ± 35.3	211.6 ± 37.0	<0.001
TG (mg/dL)	122 (87–174)	97 (72–132)	117 (87–164)	142 (104–196)	160 (117–219)	<0.001
HDL-C (mg/dL)	51.1 ± 12.0	55.6 ± 12.5	51.5 ± 11.4	48.0 ± 10.4	45.9 ± 9.6	<0.001
LDL-C (mg/dL)	131.4 ± 31.7	124.7 ± 29.6	132.6 ± 31.3	134.9 ± 31.8	139.2 ± 32.8	<0.001
AST (IU/L)	24.1 ± 13.3	21.3 ± 11.2	22.8 ± 10.8	24.5 ± 12.1	29.1 ± 16.7	<0.001
ALT (IU/L)	30.3 ± 23.7	22.1 ± 15.8	26.2 ± 19.6	33.6 ± 21.5	43.0 ± 31.3	<0.001
γ-GTP (IU/L)	30 (21–48)	23 (17–34)	31 (21–49)	33 (24–51)	43 (30–66)	<0.001
hs-CRP (mg/dL)	0.05 (0.03–0.11)	0.04 (0.02–0.07)	0.06 (0.04–0.11)^a^	0.06 (0.04–0.10)^a^	0.09 (0.05–0.16)	<0.001
HOMA-IR	1.33 (0.877–2.000)	0.982 (0.657–1.406)	1.368 (0.931–1.959)	1.506 (1.049–2.112)	2.045 (1426–2.995)	<0.001
HbA1c (%)	5.72 ± 0.55	5.60 ± 0.40	5.70 ± 0.46	5.75 ± 0.58	5.89 ± 0.70	<0.001
Waist-hip ratio	0.909 ± 0.287	0.867 ± 0.026	0.927 ± 0.026	0.882 ± 0.017	0.934 ± 0.026	<0.001
BMI (kg/m^2^)	24.7 ± 3.0	22.8 ± 2.1	26.0 ± 2.3	24.5 ± 1.9	27.1 ± 2.7	<0.001
Waist circumference (cm)	86.6 ± 7.9	81.6 ± 5.9	89.4 ± 6.1	86.7 ± 5.3	93.9 ± 7.1	<0.001
Lean mass (kg)	53.3 ± 5.8	52.0 ± 5.3^a^	52.2 ± 5.6^a^	54.4 ± 5.6	54.9 ± 6.1	<0.001
Fat mass (kg)	17.6 ± 6.0	13.7 ± 3.7	20.2 ± 4.7	17.0 ± 3.4	23.7 ± 5.8	<0.001
Percent body fat (%)	23.3 ± 5.3	19.7 ± 4.0	26.5 ± 3.8	22.7 ± 3.1	28.7 ± 4.0	<0.001
Proportion of subjects with diabetes (%)	1,264 (5.9)	229 (2.5)	130 (5.9)	303 (6.2)	602 (11.6)	<0.001
Proportion of subjects with hypertension (%)	2,116 (9.9)	496 (5.5)	290 (13.1)	424 (8.7)	907 (17.4)	<0.001
Proportion of subjects with statin medication (%)	767 (3.6)	171 (1.9)	102 (4.6)	205 (4.2)	289 (5.6)	<0.001
Proportion of who has ever smoked (%)	12,122 (56.8)	4,853 (53.6)	2,825 (58.2)	1,573 (58.5)	6,571 (60.4)	<0.001
Proportion of subjects with do regular exercise (%)	3,807 (17.8)	1,750 (19.3)	421 (19)	790 (16.3)	846 (16.3)	<0.001

Values are expressed as mean ± standard deviation, median (interquartile range) or percentage.

*NAFLD* nonalcoholic fatty liver disease, *WHR* waist-hip ratio, *BMI* body mass index, *SBP* systolic blood pressure, *DBP* diastolic blood pressure, *FBS* fasting blood glucose, *TC* total cholesterol, *TG* triglyceride, *HDL-C* high-density lipoprotein cholesterol, *LDL-C* low-density lipoprotein cholesterol, *ALT* alanine aminotransferase, *AST* aspartate aminotransferase, *HbA1c* glycated hemoglobin.

^a^No differences between the groups with same footnotes in post hoc analyses.

### Comparison of parameters between the groups according to NAFLD and abdominal obesity status

Comparison of parameters between groups revealed that the abdominal obesity only group was the oldest, and the group with both abnormalities had the worst metabolic parameters (Table 1). The metabolic parameters, with the exception of blood pressure, of the NAFLD only group were worse than those of the abdominal obesity only group. The mean WHR value of total population was 0.91, and subjects with NAFLD were generally more obese with higher mean WHR, BMI, WC, and fat mass compared to those in subjects without NAFLD (Table 1). The group with both abnormalities had the highest proportion of subjects with diabetes, hypertension, and dyslipidemia. The proportion of subjects with diabetes and dyslipidemia were higher in the NAFLD only group than in abdominal only group. However, the opposite was true for the proportion of subjects with hypertension. In addition, subjects with NAFLD tended to smoke more and exercise less than subjects without NAFLD (Table 1).

### Comparison of coronary artery calcium score among the four groups divided by non-alcoholic fatty liver disease and abdominal obesity status

**Figure 1:**
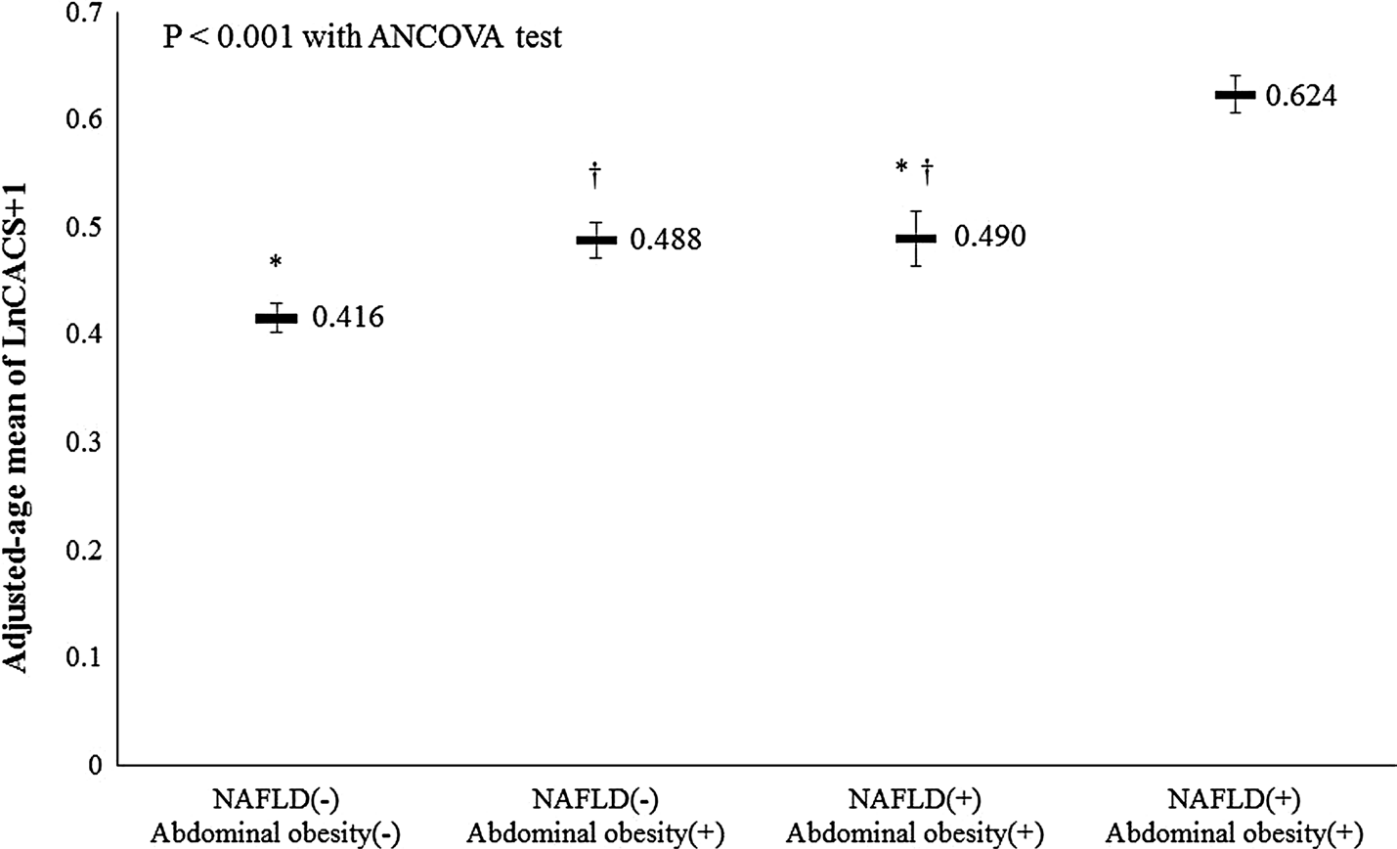
Age-adjusted means of coronary artery calcium score among the four groups. ^*,†^No differences between the groups with same footnotes in post hoc analyses.

15.9% of total population had CACS >0 and mean Ln (CACS + 1) was 0.49 (Table 2). While the mean value of CACS was the highest in the abdominal obesity only group and the lowest in the group without either abnormality, the age-adjusted mean value of CACS was the highest in the group with both abnormalities (Table 2; Figure 1). When mean Ln (CACS + 1) was compared among the groups, the mean Ln (CACS + 1) values was the highest in the abdominal obesity only group and the lowest in the NAFLD only group (Table 2). The proportion of subjects with CAC was the highest in the abdominal obesity only group and the lowest in the group without either abnormality (23.2 vs. 11.3%).

**Table 2:** Comparison of coronary artery calcium score among the four groups divided by non-alcoholic fatty liver disease and abdominal obesity status

	Total population (N = 21,335)	NAFLD (−) abdominal obesity (−) (N = 9,052)	NAFLD (−) abdominal obesity (+) (N = 2,220)	NAFLD (+) abdominal obesity (−) (N = 4,859)	NAFLD (+) abdominal obesity (+) (N = 5,204)	*p*
Mean Ln (CACS + 1)	0.49 ± 1.28	0.34 ± 1.05	0.77 ± 1.60	0.40 ± 1.16	0.72 ± 1.53	<0.001^a^
Age-adjusted mean Ln (CACS + 1)	–	0.416 ± 0.013^d^	0.490 ± 0.026^d^	0.488 ± 0.017^d^	0.624 ± 0.017	<0.001^b^
Number of subjects with CACS = 0 (%)	17,950 (84.1)	8,025 (88.7)	1,705 (76.8)	4,187 (86.2)	4,033 (77.5)	<0.001^c^
Number of subjects with CACS >0 (%)	3,385 (15.9)	1,027 (11.3)	515 (23.2)	672 (13.8)	1,171 (22.5)	<0.001^c^

*NAFLD* nonalcoholic fatty liver disease, *CACS* coronary artery calcium score.

^a^P value analyzed by one-way ANOVA.

^b^P value analyzed by ANCOVA.

^c^P value analyzed by Chi square test.

^d^No differences between the groups with same footnotes in post hoc analyses.

**Figure 2:**
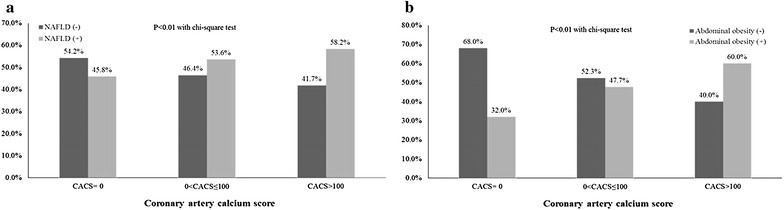
Prevalence of NAFLD (**a**) and abdominal obesity (**b**) according to the CAC grade. *NAFLD* non-alcoholic fatty liver disease, *CAC* coronary artery calcification.

Of the total subjects, 10,063 (47.2%) had NAFLD and 7,424 (34.8%) had abdominal obesity. The prevalence of NAFLD increased from 8,220 (45.8%) in subjects with CACS = 0 to 1,503 (53.6%) in subjects with 0 < CACS ≤ 100 and 340 (58.3%) in subjects with CACS >100 (Figure 2). The prevalence of abdominal obesity increased from 5,738 (32%) in subjects with CACS = 0 to 1,336 (47.7%) in subjects with 0 < CACS ≤ 100 and 794 (60%) in subjects with CACS >100 (Figure 2).

### Risk for coronary artery calcification in subjects with either NAFLD or abdominal obesity

In univariate analyses, the OR for CAC was much higher in the abdominal obesity group than in the NAFLD group. Whereas in the age adjusted model, the odds ratio (OR) of CAC in subjects with NAFLD increased compared with those without NAFLD (Table 3). OR for CAC was higher in subjects with abdominal obesity compared with those without abdominal obesity (Table 3).

**Table 3:** Odds ratio for coronary artery calcification in subjects with either NAFLD or abdominal obesity

Independent variables	Coronary artery calcification (CACS >0)								
	N (%)	Crude OR (95% CI)	*p*	Model 1 OR (95% CI)	*p*	Model 2 OR (95% CI)	*p*	Model 3 OR (95% CI)	*p*
NAFLD	1,843 (54.4)	1.415 (1.314–1.524)	<0.001	1.511 (1.395–1.637)	<0.001	1.360 (1.253–1.476)	<0.001	1.161 (1.061–1.271)	<0.001
Abdominal obesity	1,686 (49.8)	2.112 (2.097–2.656)	<0.001	1.346 (1.240–1.461)	<0.001	1.220 (1.122–1.326)	<0.001	1.005 (0.839–1.204)	0.954

Model 1 was adjusted for age.

Model 2 was adjusted for age, diabetes, hypertension, smoking and physical activity.

Model 3 was adjusted for age, diabetes, hypertension, smoking and physical activity, total cholesterol, HDL-C and HOMA-IR.

*NAFLD* non-alcoholic fatty liver disease, *CACS* coronary artery calcium score, *OR* odds ratio, *CI* confidence interval, *HDL-L* high-density lipoprotein cholesterol, *HOMA-IA* homeostasis model assessment-insulin resistance.

In multivariable analyses, after adjustment for age, diabetes history, hypertension, cigarette smoking, and physical inactivity, the OR of the NAFLD group attenuated, but the NAFLD group showed a relatively increased risk for CAC compared to those without NAFLD, and the OR was higher than that in subjects with abdominal obesity [1.360; 95% CI 1.253–1.476) vs. (1.220; 95% CI 1.122–1.326)] (Table 3). With additional adjustment for total cholesterol (TC), high-density lipoprotein cholesterol (HDL-C) and HOMA-IR, subjects with NAFLD consistently showed increased OR for CAC, whilst subjects with abdominal obesity showed non-significantly increased OR for CAC (Table 3).

### Risk for coronary artery calcification in groups divided by NAFLD and abdominal obesity status

A formal analysis to compare the association of CAC with NAFLD or abdominal obesity is shown in Table 4. In univariate analyses, the OR of CAC was the highest in the group with abdominal obesity only, the second highest in the group with both abnormalities, and the second lowest in the group with NAFLD only. In the age-adjusted model, the OR for CAC was the highest in the group with both abnormalities, and the NAFLD only group had a higher risk of CAC than the abdominal only group. Likewise, in multivariable analyses, the OR for CAC was the highest in the group with both abnormalities (1.465; 95% CI 1.324–1.623). The NAFLD only group showed significantly increased OR for CAC compared to that in the abdominal obesity only group (1.286; 95% CI 1.151–1.436) vs. (OR = 1.076; 95% CI 0.939–1.233) (Table 4). While the risk of CAC in the NAFLD only group attenuated but remained statistically significant, the abdominal obesity only group was not associated with CAC in models adjusted for age, history of diabetes, hypertension, cigarette smoking, and physical inactivity. Further adjustment for TC, HDL-C and HOMA-IR attenuated the OR with significantly increased OR for CAC only in subjects with both NAFLD and abdominal obesity (Table 4).

**Table 4:** Odds ratio for coronary artery calcification in groups divided by NAFLD and abdominal obesity status

Coronary artery calcification	Crude OR (95% CI)	*p*	Model 1 OR (95% CI)	*p*	Model 2 OR (95% CI)		Model 3 OR (95% CI)	*p*
NAFLD (−), abdominal obesity (−)	Reference	–	1.000	–	1.000	–	1.000	–
NAFLD (−), abdominal obesity (+)	2.360 (2.097–2.656)	<0.001	1.181 (1.033–1.349)	0.015	1.076 (0.939–1.233)	0.277	0.974 (0.848–1.119)	0.708
NAFLD (+), abdominal obesity (−)	1.254 (1.130–1.392)	<0.001	1.363 (1.221–1.521)	<0.001	1.286 (1.151–1.436)	<0.001	1.111 (0.990–1.247)	0.074
NAFLD (+), abdominal obesity (+)	2.269 (2.070–2.487)	<0.001	1.694 (1.535–1.870)	<0.001	1.465 (1.324–1.623)	<0.001	1.190 (1.061–1.334)	0.003

Model 1 was adjusted for age.

Model 2 was adjusted for age, diabetes, hypertension, smoking and physical activity.

Model 3 was adjusted for age, diabetes, hypertension, smoking and physical activity, total cholesterol, HDL-C and HOMA-IR.

*NAFLD* non-alcoholic fatty liver disease, *CACS* coronary artery calcium score, *OR* odds ratio, *CI* confidence interval, *HDL-L* high-density lipoprotein cholesterol, *HOMA-IA* homeostasis model assessment-insulin resistance.

## Discussion

In this study performed in a large health-screening male cohort, we found that subjects with NAFLD had a significantly increased risk for CAC compared to that in men with abdominal obesity. While the CACS was the highest in subjects with abdominal obesity only and the lowest in subjects without either abnormality, the age-adjusted mean of CACS was the highest in subjects with both abnormalities. In the post hoc analysis, the risk for CAC was the highest in subjects with both abnormalities, the subjects with NAFLD without abdominal obesity had a significantly increased risk for CAC, and the subjects with abdominal obesity without NAFLD were not significantly associated with CAC. Our results indicate that NAFLD is more closely associated with coronary artery calcification than abdominal obesity.

NAFLD refers to the accumulation of fat in more than 5% of hepatocytes in subjects whose alcohol intake is lower than 20 g/d [6]. Although NAFLD is known as the hepatic manifestation of IR or metabolic syndrome, more recent reports have suggested that subjects with NAFLD are at increased risk for CAD independent of metabolic syndrome and insulin resistance [33, 34]. Many studies have reported increased risk for CAC in subjects with NAFLD [17–23]. Kim et al. reported that NAFLD is associated with CAC independently of the traditional risk factors, including visceral adipose tissue [35]. Further researches suggest that not just NAFLD, but the presence of non-alcoholic steatohepatitis (NASH) or fibrosis is more associated with development of atherosclerosis [36, 37]. Meanwhile, obesity has a variable association with CAC depending on the measures used for comparison [38]. The most recent reports indicate that when abdominal obesity is assessed using the WHR, it can be considered a strong risk factor for CAC [39–41]. Our research shows that abdominal obesity measured using the WHR was highly correlated with CAC across the body.

In this study, men with WHR greater than 0.9 and without NAFLD were older and had higher blood pressure than men with NAFLD and without abdominal obesity. Other studies have reported that the WHR is closely related to hypertension [42, 43]. Our results illustrate that CACS was highest in subjects with only abdominal obesity. After adjusting for risk factors of CAD including age and metabolic parameters, NAFLD and abdominal obesity showed a synergistically increased risk for subclinical atherosclerosis. In addition, subjects with NAFLD only retained a significantly higher risk for CAC than those with only abdominal obesity when other well-established risk factors were taken into account. One of the reasons for the higher risk for CAC in subjects with NAFLD only, could be due to the higher prevalence of diabetes in subjects with NAFLD only compared with those with abdominal obesity only, since, diabetes is a well-known cause for CAC [44]. However, the significantly increased risk for CAC was consistent even after adjustment for the presence of diabetes. Our result was in the opposite to the result from Coronary Artery Risk Development in Young Adults (CARDIA) Study performed in 2,424 participants, in that the association of CAC and NAFLD was attenuated after additional adjustment for visceral adiposity, which suggest the importance of abdominal obesity in association between CAC and NAFLD [20].

Fat accumulation in hepatocytes is more closely associated with VAT [45]. Increased VAT plays a role in the pathogenesis of NAFLD due to the secretion of pro-inflammatory cytokines and adipokines, and the release of free fatty acids into the portal system [46, 47]; thus visceral fat accumulation might be a mediator that links NAFLD to CAD [48]. A recent review and a study has shown that inflammation may be an underlying cause of CAD and also CAC [18, 49]. However, in our study, there was no significant difference in the inflammatory marker hs-CRP between the NAFLD only and abdominal obesity only groups. Several researchers have reported that abdominal obesity is consistently associated with NAFLD in 60–95% of the cases, and abdominal obesity assessed by WHR is closely related the occurrence of NAFLD [50]. This means that a high WHR, without evidence of NAFLD, may be limited as an independent risk factor for CAD.

The present study has some limitations. First, MDCT for CAC scoring was performed as part of a health checkup rather than for any specified research purpose, and there were no predetermined criteria for undergoing the test. Most of the examinees were employees and family members of various industrial companies from around the country. Second, it has been reported that ultrasonography has high and variable sensitivity and cannot distinguish steatohepatitis from simple steatosis [51]. In addition, the failure to perform transient elastography is a major limitation in our study, since a recent study reported the independent association of hepatic fibrosis assessed by elastography with CACS [21]. Third, determination of alcohol intake solely by self-questionnaire might have caused bias. Lastly, the cross-sectional nature of the study design cannot contribute to clarifying how mechanistically NAFLD is associated with CAC.

## Conclusion

In this large, health-screening population, CACS was significantly higher in subjects with NAFLD than in those with abdominal obesity after adjusting for age, diabetes, hypertension, smoking, and physical activity, including some of the components involved with metabolic syndrome. Very little is known about the exact role of NAFLD as a risk factor for CAD. However, it is clear that the prevalence of NAFLD has greatly increased in recent years, and that of CAD has increased even in subjects without abdominal obesity. Therefore, it is important to find the underlying pathogenesis that links NAFLD to CAD, and to identify the necessary management to prevent and reduce the causes of these two diseases. In the future diagnosis of NAFLD, particular attention should be focused on men who are most likely to benefit from intensive lifestyle modification and pharmacological treatment to decrease CAD risk.

## References

[1] Mendis S, Puska P, Norrving B (2011) Global atlas on cardiovascular disease prevention and control. World Health Organization in collaboration with the World Heart Federation and World Stroke Organization, Geneva

[2] Dahlén EM, Bjarnegård N, Länne T, Nystrom FH, Ostgren CJ (2013). Sagittal abdominal diameter is a more independent measure compared with waist circumference to predict arterial stiffness in subjects with type 2 diabetes—a prospective observational cohort study. Cardiovasc Diabetol..

[3] Ren C, Zhang J, Xu Y, Xu B, Sun W, Sun J (2014). Association between carotid intima-media thickness and index of central fat distribution in middle-aged and elderly Chinese. Cardiovasc Diabetol.

[4] Hubert HB, Feinleib M, McNamara PM, Castelli WP (1983). Obesity as an independent risk factor for cardiovascular disease: a 26-year follow-up of participants in the Framingham Heart Study. Circulation.

[5] Osawa K, Miyoshi T, Koyama Y, Sato S, Akagi N, Morimitsu Y (2014). Differential association of visceral adipose tissue with coronary plaque characteristics in patients with and without diabetes mellitus. Cardiovasc Diabetol.

[6] Lakka TA, Lakka HM, Salonen R, Kaplan GA, Salonen JT (2001). Abdominal obesity is associated with accelerated progression of carotid atherosclerosis in men. Atherosclerosis.

[7] Rexrode KM, Carey VJ, Hennekens CH, Walters EE, Colditz GA, Stampfer MJ (1998). Abdominal adiposity and coronary heart disease in women. JAMA.

[8] Marchesini G, Brizi M, Bianchi G, Tomassetti S, Bugianesi E, Lenzi M (2001). Nonalcoholic fatty liver disease: a feature of the metabolic syndrome. Diabetes.

[9] Fraser A, Longnecker MP, Lawlor DA (2007). Prevalence of elevated alanine aminotransferase among US adolescents and associated factors: NHANES 1999-2004. Gastroenterology.

[10] Solymoss BC, Bourassa MG, Campeau L, Sniderman A, Marcil M, Lespérance J (2004). Effect of increasing metabolic syndrome score on atherosclerotic risk profile and coronary artery disease angiographic severity. Am J Cardiol.

[11] Villanova N, Moscatiello S, Ramilli S, Bugianesi E, Magalotti D, Vanni E (2005). Endothelial dysfunction and cardiovascular risk profile in nonalcoholic fatty liver disease. Hepatology.

[12] Fargion S, Porzio M, Fracanzani AL (2014). Nonalcoholic fatty liver disease and vascular disease: state-of-the-art. World J Gastroenterol.

[13] Puchner SB, Lu MT, Mayrhofer T, Liu T, Pursnani A, Ghoshhajra BB (2015). High-risk coronary plaque at coronary CT angiography is associated with nonalcoholic fatty liver disease, independent of coronary plaque and stenosis burden: results from the ROMICAT II trial. Radiology.

[14] Wexler L, Brundage B, Crouse J, Detrano R, Fuster V, Maddahi J (1996). Coronary artery calcification: pathophysiology, epidemiology, imaging methods, and clinical implications. A statement for health professionals from the American Heart Association. Writing Group. Circulation.

[15] Rumberger JA, Simons DB, Fitzpatrick LA, Sheedy PF, Schwartz RS (1995). Coronary artery calcium area by electron-beam computed tomography and coronary atherosclerotic plaque area. A histopathologic correlative study. Circulation.

[16] Budoff MJ, Achenbach S, Blumenthal RS, Carr JJ, Goldin JG, Greenland P (2006). American Heart Association Committee on Cardiovascular Imaging and Intervention; American Heart Association Council on Cardiovascular Radiology and Intervention; American Heart Association Committee on Cardiac Imaging, Council on Clinical Cardiology. Assessment of coronary artery disease by cardiac computed tomography: a scientific statement from the American Heart Association Committee on Cardiovascular Imaging and Intervention, Council on Cardiovascular Radiology and Intervention, and Committee on Cardiac Imaging, Council on Clinical Cardiology. Circulation.

[17] Chhabra R, O’Keefe JH, Patil H, O’Keefe E, Thompson RC, Ansari S (2013). Association of coronary artery calcification with hepatic steatosis in asymptomatic individuals. Mayo Clin Proc.

[18] Al Rifai M, Silverman MG, Nasir K, Budoff MJ, Blankstein R, Szklo M (2015). The association of nonalcoholic fatty liver disease, obesity, and metabolic syndrome, with systemic inflammation and subclinical atherosclerosis: the Multi-Ethnic Study of Atherosclerosis (MESA). Atherosclerosis.

[19] Mellinger JL, Pencina KM, Massaro JM, Hoffmann U, Seshadri S, Fox CS et al (2015) Hepatic steatosis and cardiovascular disease outcomes: an analysis of the Framingham Heart Study. J Hepatol. doi:10.1016/j.jhep.2015.02.045 10.1016/j.jhep.2015.02.045 PMC5282653 25776891

[20] VanWagner LB, Ning H, Lewis CE, Shay CM, Wilkins J, Carr JJ (2014). Associations between nonalcoholic fatty liver disease and subclinical atherosclerosis in middle-aged adults: the Coronary Artery Risk Development in Young Adults Study. Atherosclerosis.

[21] You SC, Kim KJ, Kim SU, Kim BK, Park JY, Kim DY et al (2015) Hepatic fibrosis assessed using transient elastography is independently associated with coronary artery calcification. J Gastroenterol Hepatol. doi:10.1111/jgh.12992 10.1111/jgh.12992 25973647

[22] Kwak MS, Yim JY, Kim D, Park MJ, Lim SH, Yang JI (2015). Nonalcoholic fatty liver disease is associated with coronary artery calcium score in diabetes patients with higher HbA1c. Diabetol Metab Syndr.

[23] Sung KC, Lim YH, Park S, Kang SM, Park JB, Kim BJ (2013). Arterial stiffness, fatty liver and the presence of coronary artery calcium in a large population cohort. Cardiovasc Diabetol.

[24] Curb JD, Ford C, Hawkins CM, Smith EO, Zimbaldi N, Carter B (1983). A coordinating center in a clinical trial: the Hypertension Detection and Followup Program. Control Clin Trials.

[25] Matthews DR, Hosker JP, Rudenski AS, Naylor BA, Treacher DF, Turner RC (1985). Homeostasis model assessment: insulin resistance and beta-cell function from fasting plasma glucose and insulin concentrations in man. Diabetologia.

[26] NGSP (2010) List of NGSP certified methods: NSGP (updated 2013 Feb 1; cited 2014 Aug 4). http://www.ngsp.org/docs/methods.pdf

[27] Standards of medical care in diabetes (2015) Summary of revisions. Diabetes Care 38(Suppl:S4). doi:10.2337/dc15-S003 10.2337/dc15-S003 25537706

[28] Chobanian AV, Bakris GL, Black HR, Cushman WC, Green LA, Izzo JL (2003). Joint National Committee on Prevention, Detection, Evaluation, and Treatment of High Blood Pressure. National Heart, Lung, and Blood Institute; National High Blood Pressure Education Program Coordinating Committee. The Seventh Report of the Joint National Committee on Prevention, Detection, Evaluation, and Treatment of High Blood Pressure: the JNC 7 report. JAMA.

[29] Agatston AS, Janowitz WR, Hildner FJ, Zusmer NR, Viamonte M, Detrano R (1990). Quantification of coronary artery calcium using ultrafast computed tomography. J Am Coll Cardiol.

[30] Saverymuttu SH, Joseph AE, Maxwell JD (1986). Ultrasound scanning in the detection of hepatic fibrosis and steatosis. BMJ.

[31] Yajima Y, Ohta K, Narui T, Abe R, Suzuki H, Ohtsuki M (1983). Ultrasonographical diagnosis of fatty liver: significance of the liver-kidney contrast. Tohoku J Exp Med.

[32] Yusuf S, Hawken S, Ounpuu S, Dans T, Avezum A, Lanas F (2004). Effect of potentially modifiable risk factors associated with myocardial infarction in 52 countries (the INTERHEART study): case-control study. Lancet.

[33] Targher G, Bertolini L, Rodella S, Tessari R, Zenari L, Lippi G (2007). Nonalcoholic fatty liver disease is independently associated with an increased incidence of cardiovascular events in type 2 diabetic patients. Diabetes Care.

[34] Sung KC, Wild SH, Kwag HJ, Byrne CD (2012). Fatty liver, insulin resistance, and features of metabolic syndrome: relationships with coronary artery calcium in 10,153 people. Diabetes Care.

[35] Kim D, Choi SY, Park EH, Lee W, Kang JH, Kim W (2012). Nonalcoholic fatty liver disease is associated with coronary artery calcification. Hepatology.

[36] Petta S, Argano C, Colomba D, Cammà C, Di Marco V, Cabibi D (2015). Epicardial fat, cardiac geometry and cardiac function in patients with non-alcoholic fatty liver disease: association with the severity of liver disease. J Hepatol.

[37] Petta S, Torres D, Fazio G, Cammà C, Cabibi D, Di Marco V (2012). Carotid atherosclerosis and chronic hepatitis C: a prospective study of risk associations. Hepatology.

[38] Cassidy AE, Bielak LF, Zhou Y, Sheedy PF, Turner ST, Breen JF (2005). Progression of subclinical coronary atherosclerosis: does obesity make a difference?. Circulation.

[39] See R, Abdullah SM, McGuire DK, Khera A, Patel MJ, Lindsey JB (2007). The association of differing measures of overweight and obesity with prevalent atherosclerosis: the Dallas Heart Study. J Am Coll Cardiol.

[40] Lee CD, Jacobs DR, Schreiner PJ, Iribarren C, Hankinson A (2007). Abdominal obesity and coronary artery calcification in young adults: the Coronary Artery Risk Development in Young Adults (CARDIA) Study. Am J Clin Nutr.

[41] Yu JH, Yim SH, Yu SH, Lee JY, Kim JD, Seo MH (2013). The relationship of body composition and coronary artery calcification in apparently healthy Korean adults. Endocrinol Metab (Seoul).

[42] Feldstein CA, Akopian M, Olivieri AO, Kramer AP, Nasi M, Garrido D (2005). A comparison of body mass index and waist-to-hip ratio as indicators of hypertension risk in an urban Argentine population: a hospital-based study. Nutr Metab Cardiovasc Dis..

[43] Nemesure B, Wu SY, Hennis A (2008). Leske MC; BES Study Group. The relationship of body mass index and waist-hip ratio on the 9-year incidence of diabetes and hypertension in a predominantly African-origin population. Ann Epidemiol.

[44] Snell-Bergeon JK, Budoff MJ, Hokanson JE (2013). Vascular calcification in diabetes: mechanisms and implications. Curr Diabetes Rep.

[45] Thamer C, Machann J, Haap M, Stefan N, Heller E, Schnödt B (2004). Intrahepatic lipids are predicted by visceral adipose tissue mass in healthy subjects. Diabetes Care.

[46] Silverman JF, O’Brien KF, Long S, Leggett N, Khazanie PG, Pories WJ (1990). Liver pathology in morbidly obese patients with and without diabetes. Am J Gastroenterol.

[47] Milic S, Lulic D, Stimac D (2014). Non-alcoholic fatty liver disease and obesity: biochemical, metabolic and clinical presentations. World J Gastroenterol.

[48] Targher G, Bertolini L, Padovani R, Zenari L, Zoppini G, Falezza G (2004). Relation of nonalcoholic hepatic steatosis to early carotid atherosclerosis in healthy men: role of visceral fat accumulation. Diabetes Care.

[49] Hansson GK (2005). Inflammation, atherosclerosis, and coronary artery disease. New Engl J Med.

[50] Zheng RD, Chen ZR, Chen JN, Lu YH, Chen J (2012). Role of body mass index, waist-to-height and waist-to-hip ratio in prediction of nonalcoholic fatty liver disease. Gastroenterol Res Pract.

[51] Mottin CC, Moretto M, Padoin AV, Swarowsky AM, Toneto MG, Glock L (2004). The role of ultrasound in the diagnosis of hepatic steatosis in morbidly obese patients. Obes Surg.

